# Atrial fibrillation and the risk of myocardial infarction: a nation-wide propensity-matched study

**DOI:** 10.1038/s41598-017-13061-4

**Published:** 2017-10-05

**Authors:** Hye Young Lee, Pil-Sung Yang, Tae-Hoon Kim, Jae-Sun Uhm, Hui-Nam Pak, Moon-Hyoung Lee, Boyoung Joung

**Affiliations:** 1 Division of Cardiology, Yonsei University College of Medicine, Seoul, Korea; 2 Division of Cardiology, Sanggye Paik Hospital, Inje University College of Medicine, Seoul, Korea

## Abstract

In addition to being an established complicating factor for myocardial infarction (MI), recent studies have revealed that atrial fibrillation (AF) increased risk of MI. This study is to evaluate the risk of MI associated with AF in a nationwide population based cohort. We examine the association between AF and incident MI in 497,366 adults from the Korean National Health Insurance Service database, who were free of AF and MI at baseline. AF group (n = 3,295) was compared with propensity matched no-AF group (n = 13,159). Over 4.2 years of follow up, 137 MI events occurred. AF was associated with 3-fold increased risk of MI (HR, 3.1; 95% CI, 2.22–4.37) in both men (HR, 2.91; 95% CI 1.91–4.45) and women (HR, 3.52; 95% CI 2.01–6.17). The risk of AF-associated MI was higher in patients free of hypertension, diabetes, ischemic stroke, and dyslipidemia at baseline. The cumulative incidence of AF-associated MI was lower in patients on anticoagulant and statin therapies. Our finding suggests that AF complications beyond stoke should extend to total mortality to include MI.

## Introduction

The significance of atrial fibrillation (AF) as a major public health problem comes from its increasing prevalence and strong association with morbidity and mortality^1,2^. Patients with AF have 5 times the risk of stroke and double the risk of mortality compared with those without AF^3,4^. AF has known to complicate acute myocardial infarction (MI)^5^. In addition to being an established complicating factor for MI, recent studies have revealed that AF increased the risk of MI^6,7^. In the Atherosclerosis Risk in Communities (ARIC) study, AF was associated with an increased risk of non-ST segment elevation MI (NSTEMI)^6^. In a recent study performed by Soliman *et al*.^7^, AF was associated with a 70% increased risk of incident MI. However, these results are yet to be validated in a nationwide population based cohort and the mechanisms explaining these associations are not fully understood.

Thus, we examined the association between AF and MI by analyzing a recently developed Korean National Health Insurance Service–national sample cohort (NHIS-NSC) database, which includes over five hundred thousand individuals. In addition, the beneficial effects of the commonly prescribed medications for AF patients on the occurrence of MI have not been elucidated. Herein, we also analyzed the association of medications with incident MI in AF patients.

## Methods

### Source of study data

The national health insurance service (NHIS) in Korea is a single-payer program and is mandatory for all residents in South Korea. This retrospective study used the NHIS-NSC 2002–2013 dataset, comprising a random sample of 1,025,340 subjects, which amounted to 2.2% of the entire Korean population in the NHIS in 2002 (46,605,433). Data were produced by the NHIS using a systematic sampling method for the purpose of research. Random sampling was used based on 1,476 strata^8^. As all individuals in South Korea are enrolled in the NHIS, this database represents the entire Korean population^9^. This is a semi-dynamic cohort database; the cohort has been followed up to either the time of the participant’s disqualification of health services due to death or emigration or the end of the study period. The database contains eligibility and demographic information regarding health insurance and medical aid beneficiaries, medical bill details, diagnostic codes, medical treatment, disease histories, prescription drug used, and personal information for inpatients and outpatient visits.

In this cohort, the subjects’ disease information was classified according to the 10^th^ revision of the International Classification of Diseases (ICD-10) codes obtained from the Korean National Statistical Office (Supplementary Table S1). This study was approved by the Institutional Review Board (IRB) of Yonsei University College of Medicine in Seoul, Korea. The IRB waived the requirement to obtain informed consent, and this study was conducted in accordance with the tenets of the Declaration of Helsinki.

### Study population

A total of 506,805 patients, who had a health check-up between 2009 and 2013, were enrolled and follow-up data were reviewed until December 2013. AF cases were identified by ICD-10 codes (ICD-10: I48)^10^. To ensure accuracy of diagnosis, we defined patients as AF only when it was a discharge diagnosis or was confirmed more than twice in the outpatient department. To further evaluate the accuracy of the definition of AF, a validation study was performed in 628 randomly selected patients with ICD-10 code of I48 in 2 separate hospitals. Their electrocardiograms (ECGs) were reviewed by two physicians. Patients were determined to have AF if documented by ECG. The positive predictive value was 94.1%. The clinical end point was the first occurrence of MI during follow up. MI cases were identified by ICD-10 codes of I21or I22, and were ascertained when the diagnosis was confirmed by hospitalization or death from MI. To evaluate the accuracy of our definition of MI, we conducted a validation study with medical records of two independent tertiary hospitals from 2006–2013. A total of 4,688 patients were found to have ICD codes of I21 or I22. Data of clinical history, cardiac biomarkers, ECGs and the results of coronary angiography were reviewed by two cardiologists. The positive predictive value was 86.5%.

Medical records of all patients were reviewed from 2002 till 2013 in order to clarify the newly diagnosed AF and MI during the study period. The laboratory and survey questionnaire data of general and life-transition health examinations for all cohort members were merged.

Of the 8,296 patients with AF, patients who had AF or MI before 2009 (n = 4,976) and who suffered an MI before AF (n = 25) were excluded. Finally 3,295 patients were enrolled in the AF group. Among those without AF (n = 498,509), 494,071 patients were enrolled in the no-AF group after excluding the patients who suffered an MI before 2009 (n = 4,438). Given the differences in the baseline characteristics and the risk of cardiovascular diseases between the AF and control groups, we used 1:4 propensity score matching and calculated the propensity score, with predicted probability of AF occurrence conditional on baseline covariates, by multivariable logistic regression (Table 1)^11^. We attempted to match each patient in the AF cohort with a patient in the control cohort with a similar propensity score, based on nearest-neighbor matching without replacement, using a caliper width equal to 0.05 of the standard deviation of the logit of the propensity score. Pre- and post-match absolute standardized differences were presented as Love Plots^12^.

**Table 1:** Baseline characteristics by AF status before and after propensity score matching

	Before propensity matching			p	After propensity matching			p
	No AF (n = 494,071)	AF (n = 3,295)	Standard mean, Diff.		No AF (n = 13,159)	AF (n = 3,295)	Standard, mean, Diff.	
Age, years	47.4 ± 14.2	62.8 ± 12.9	1.194	< 0.001	62.9 ± 12.9	62.8 ± 12.9	−0.006	0.877
Female, %	50.1	42	−0.173	< 0.001	42.1	42.0	−0.003	0.874
Systolic BP, mmHg	122.1 ± 15.3	128.3 ± 16.9	0.366	< 0.001	128.5 ± 16.1	128.3 ± 16.8	−0.012	0.577
Diastolic BP, mmHg	76.0 ± 10.2	78.6 ± 10.6	0.245	< 0.001	78.8 ± 10.2	78.6 ± 10.7	−0.012	0.586
Body mass index, kg/m2	23.7 ± 3.3	24.3 ± 3.4	0.190	< 0.001	24.3 ± 3.2	24.3 ± 3.4	0.003	0.884
Waist circumference, cm	79.9 ± 9.3	83.9 ± 9.1	0.449	< 0.001	84.0 ± 8.6	84.0 ± 9.1	0.003	0.629
Heart failure, %	1.9	11.5	0.299	< 0.001	10.1	11.5	0.038	0.023
Hypertension, %	20.7	57	0.732	< 0.001	56.5	57	0.009	0.609
Diabetes, %	12.3	32.1	0.426	< 0.001	31.3	32.1	0.017	0.345
CKD or ESRD, %	5.6	17.3	0.309	< 0.001	16.6	17.3	0.015	0.348
Dyslipidemia, %	18.6	38.1	0.400	< 0.001	38.2	38.1	−0.003	0.952
Ischemic stroke, %	2.4	9.3	0.239	< 0.001	9.1	9.3	0.005	0.737
PAOD, %	6.9	19.4	0.315	< 0.001	19.2	19.4	0.005	0.768
COPD, %	6.0	17.3	0.298	< 0.001	16.6	17.3	0.015	0.355
History of malignancy, %	6.6	14.1	0.218	< 0.001	13.7	14.1	0.011	0.553
Anemia, %	11.8	16.7	0.245	< 0.001	16.3	16.2	−0.004	0.873
Smoking, %	37.6	39.4	0.021	0.042	38.4	39.4	0.004	0.304
Total cholesterol, mg/dl	195.1 ± 37.2	192.9 ± 39.4	−0.054	0.001	192.9 ± 37.8	192.9 ± 39.3	0.002	0.876
Triglyceride, mg/dl	131.9 ± 94.1	139.2 ± 88	0.088	< 0.001	139.6 ± 86.7	139.2 ± 88.2	−0.004	0.799
HDL-cholesterol, mg/dl	56.5 ± 27.9	55.1 ± 36.5	−0.028	0.025	54.8 ± 35.3	55.1 ± 36.5	0.006	0.721
LDL-cholesterol, mg/dl	113.8 ± 37.6	112.4 ± 38.7	−0.031	0.038	112.8 ± 38.8	112.4 ± 38.7	−0.007	0.553
Serum creatinine, mg/dl	1.0 ± 1.1	1.1 ± 1.2	0.079	< 0.001	1.09 ± 1.2	1.10 ± 1.2	0.007	0.693
eGFR-CKD-EPI	89.5 ± 21.1	76.7 ± 21.1	−0.601	< 0.001	77.4 ± 20.5	77.1 ± 20.9	−0.010	0.507
CHADS_2_	0.5 ± 0.9	1.4 ± 1.4	0.995	< 0.001	1.4 ± 1.4	1.4 ± 1.4	0.029	0.131
CHADS-VASc	1.2 ± 1.3	2.6 ± 1.9	1.073	< 0.001	2.5 ± 2.0	2.6 ± 2.0	0.026	0.225

Data are presented as mean ± SD for continuous variables and as proportions for categorical variables. AF, atrial fibrillation; CKD, chronic kidney disease; ESRD, end–stage renal disease; PAOD, peripheral artery obstructive disease; COPD, chronic obstructive pulmonary disease; HDL, high-density lipoprotein; LDL, low-density lipoprotein; eGFR-CKD-EPI, estimated glomerular filtration rate-chronic kidney disease-epidemiology collaboration; CHADS_2_, congestive heart failure, hypertension, age ≥ 75 years, diabetes mellitus, stroke; VASc, Vascular disease, age 65–75 years, sex category.

**Figure 1:**
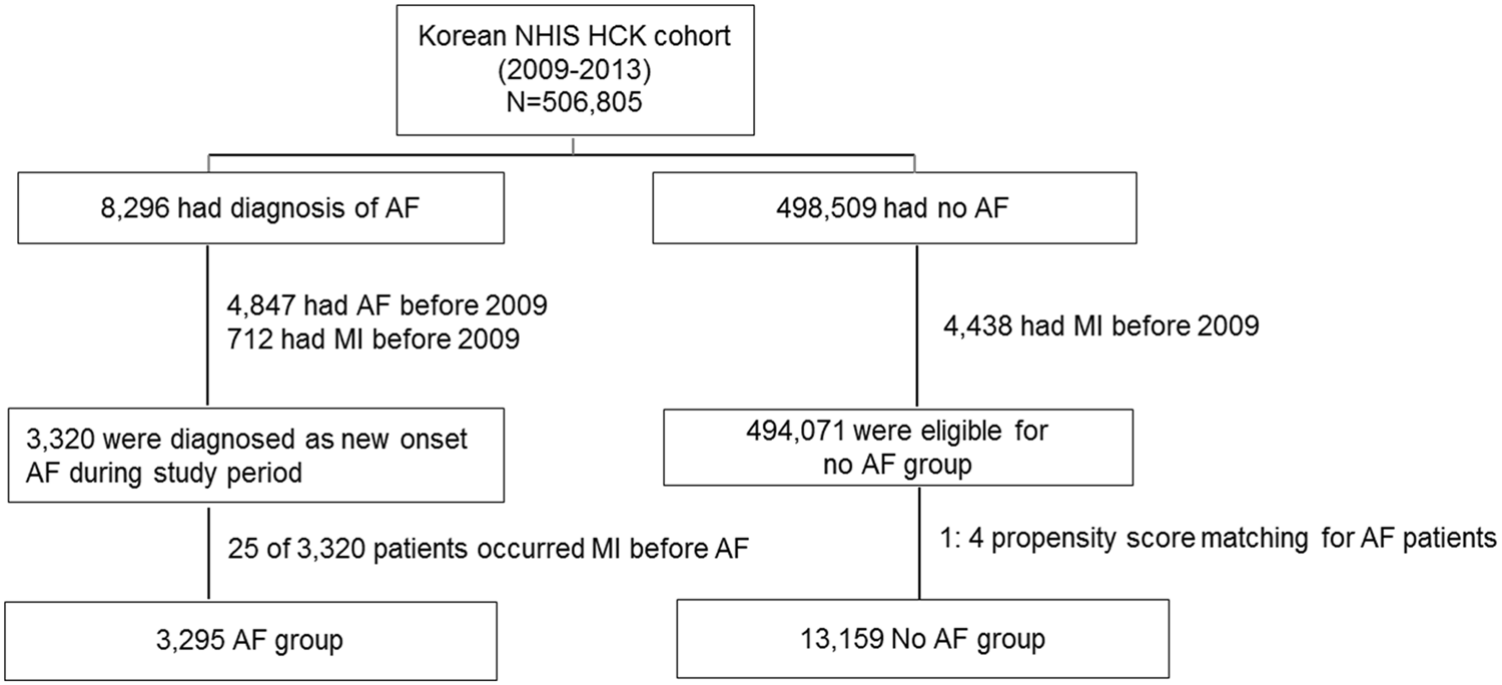
Study population. NHIS HCK, National Health Insurance Service-health check-up; AF, atrial fibrillation; MI, myocardial infarction.

The following variables were entered: age, sex, and a history of congestive heart failure, hypertension, diabetes mellitus, chronic kidney disease (CKD) or end-stage renal disease (ESRD), dyslipidemia, ischemic stroke, peripheral artery occlusive disease (PAOD), chronic obstructive pulmonary disease (COPD), history of malignancy, anemia, smoking history, blood pressure, body mass index, waist circumference, body surface area, cholesterol level, serum creatinine, and estimated –glomerular filtration rate (eGFR). Covariates included in propensity matching were the data from baseline examination at the time of health check-up, and the definitions of comorbidities were based on ICD codes. The matching procedure was performed using R packages (R Foundation for Statistical Computing, Vienna, Austria), including Matchit, RItools, and CEM^13^. Finally, a total of 3,295 AF patients and 13,159 no-AF patients were evaluated in this study (Fig. 1).

### Statistical analysis

Data were presented as mean ± SD for normally distributed continuous variables and as proportions for categorical variables. Age-adjusted incidence rate of MI per 1000 person-years in patients with and without AF was calculated in the propensity matched population and in the sex subgroup. Person-time for the incidence rate in the AF group was calculated from AF diagnosis to the occurrence of MI or censoring. In the no-AF group, person-time was calculated from baseline to MI occurrence or censoring. Cumulative incidence of MI was plotted using a Kaplan-Meier method, with statistical significance examined using log-rank test by AF status. The risk of MI was assessed using Cox regression analysis. Statistical analysis was performed using SPSS 21.0 statistical software (SPSS Inc., Chicago, IL, USA). All p-values were two tailed, and values less than 0.05 were considered statistically significant.

## Results

### Baseline characteristics

The mean age of matched patients was 63 years (range, 19–98 years) and 58% of the patients were male. Table 1 shows the baseline characteristics of the study population stratified by AF status. In the full pre-match cohort, patients with AF were older, had a higher body mass index, a higher prevalence of heart failure (HF), hypertension, diabetes, CKD or ESRD, dyslipidemia, ischemic stroke, PAOD, and COPD (all P < 0.001). Mean CHA_2_DS_2_-VASc score was 2.6 ± 1.9 in the AF group and 1.2 ± 1.3 in the no-AF group (P < 0.001). Covariates with significant imbalances in the pre-matched cohort which were well balanced after propensity matching are displayed in Table 1. Absolute standardized differences in all measured covariates were less than 5%, suggesting substantial covariate balance across the groups (Supplementary Fig. S1).

Aspirin, anticoagulants, statin, digoxin, beta blocking agent (BB), and calcium channel blocker (CCB) were more frequently prescribed in the AF group than in the no-AF group; aspirin (50.3% vs. 27.5%, P < 0.001), anticoagulant (20.9% vs. 0.3%, P < 0.001), statin (38.2% vs. 28.5%, P < 0.001), digoxin (13.4% vs. 0.9%, P < 0.001), BB (43.1% vs.16.1%, P < 0.001), and CCB (11.4% vs. 2.5%, P < 0.001).

### AF and the risk of MI

**Figure 2:**
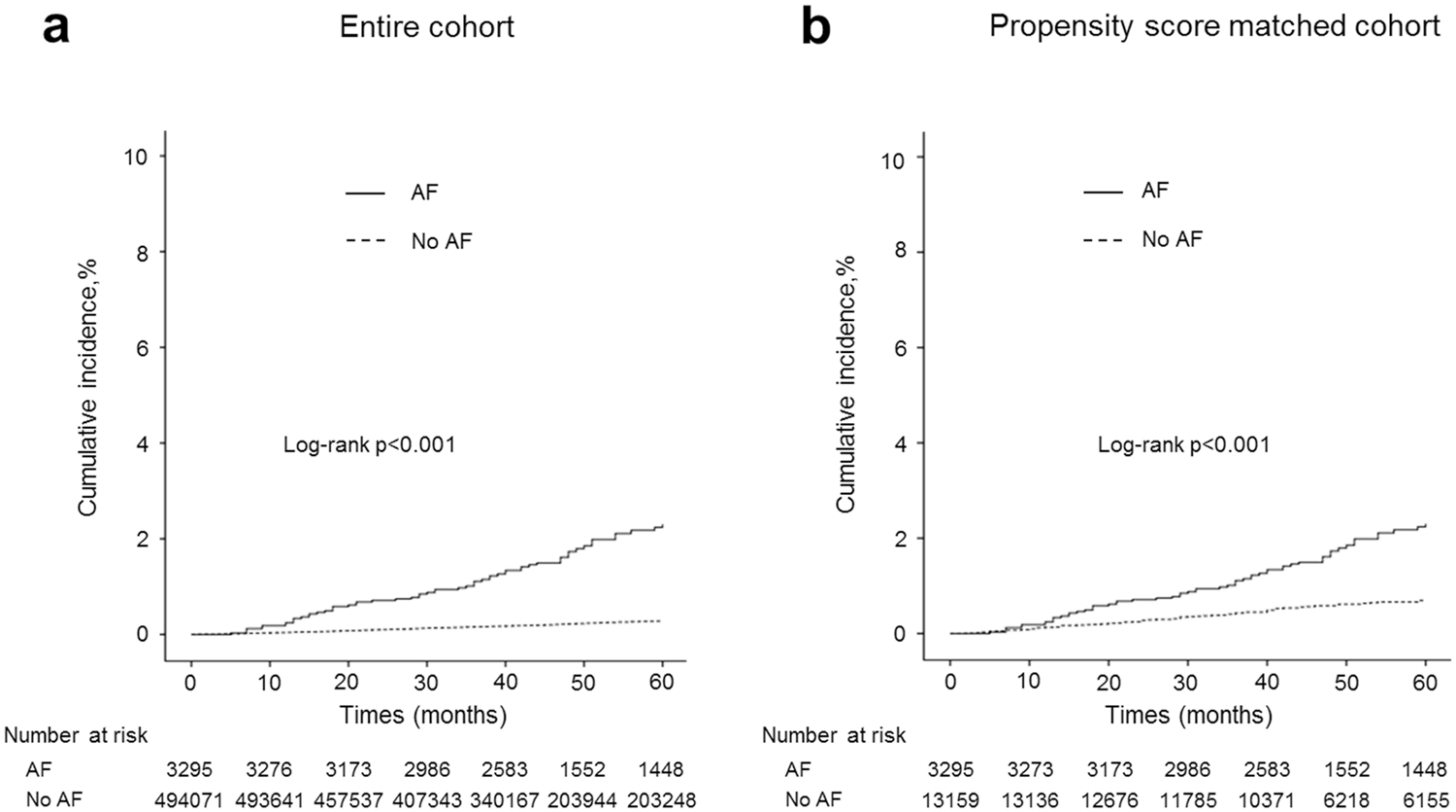
Unadjusted cumulative incidence of myocardial infarction by baseline atrial fibrillation status in the entire cohort (**a**) and in the propensity scored matched cohort (**b**). Cumulative incidence was calculated using Kaplan-Meier estimates and compared using the log-rank test.

Over 4.2 years of follow up, 137 incident MI events occurred. Figure 2 shows the unadjusted cumulative incidence of MI stratified by baseline AF status in the entire cohort (Fig. 2a) and the matched cohort (Fig. 2b). Patients with AF had a higher incidence of MI than those without AF in both the entire cohort and the matched cohort (log rank p < 0.001 for both cohorts).

**Figure 3:**
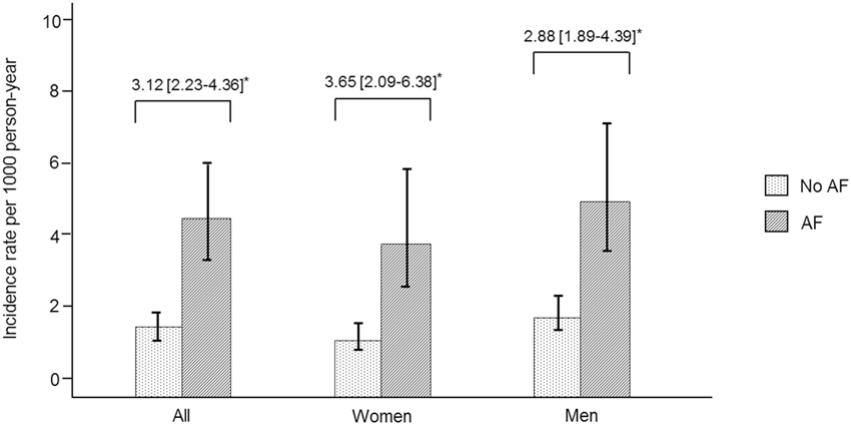
Age-adjusted incidence rate and incidence rate ratios of myocardial infarction by atrial fibrillation status. Incidence rate per 1,000 person-years. *Age adjusted incidence rate and incidence rate ratios were based on the average of the cohort.

The age-adjusted incidence rate of MI among participants was higher in those with AF than in those without AF (4.42 vs 1.42 per 1000 person-years), with an incidence rate ratio (IRR) of 3.12 [95% CI, 2.23–4.36]. In a sex stratified analysis, the higher age-adjusted MI IRRs by AF status was observed in women (IRR, 3.65; 95% CI, 2.09–6.38) compared with men (IRR, 2.88; 95% CI, 1.89–4.39) (Fig. 3).

In a Cox proportional hazards model of the matched cohort, AF [Hazard ratio (HR), 3.12; 95% CI, 2.23–4.37, p < 0.001] was an independent risk factor for MI. Other risk factors were hypertension (HR, 1.87; 95% CI, 1.30–2.69, p < 0.001), diabetes (HR, 1.79; 95% CI, 1.28–2.51, p < 0.001), HF (HR, 2.04; 95% CI, 1.32–3.14, p = 0.003), CKD or ESRD (HR, 2.70; 95% CI, 1.90–3.85, p < 0.001) and dyslipidemia (HR, 1.45; 95% CI, 1.04–2.03, p = 0.031) (Table 2).

**Table 2:** The independent clinical predictors of myocardial infarction in propensity score matched cohort.

	HR	95% CI	p-value
Age, per 1 year	1.05	1.03–1.07	< 0.001
Atrial fibrillation	3.12	2.23–4.37	< 0.001
Heart failure	2.04	1.32–3.14	0.003
Hypertension	1.87	1.30–2.69	< 0.001
Diabetes	1.79	1.28–2.51	< 0.001
CKD or ESRD	2.70	1.90–3.85	< 0.001
Dyslipidemia	1.45	1.04–2.03	0.031
Ischemic stroke	1.71	1.05–2.77	0.030
COPD	2.18	1.51–3.15	< 0.001
Anemia	1.54	1.02–2.30	0.038
Waist circumference	1.03	1.01–1.05	0.011

HR, hazard ratio; CI, confidence interval; CKD, chronic kidney disease; ESRD, end stage renal disease; COPD, chronic obstructive pulmonary disease

### Association of AF with MI in subgroups

**Figure 4:**
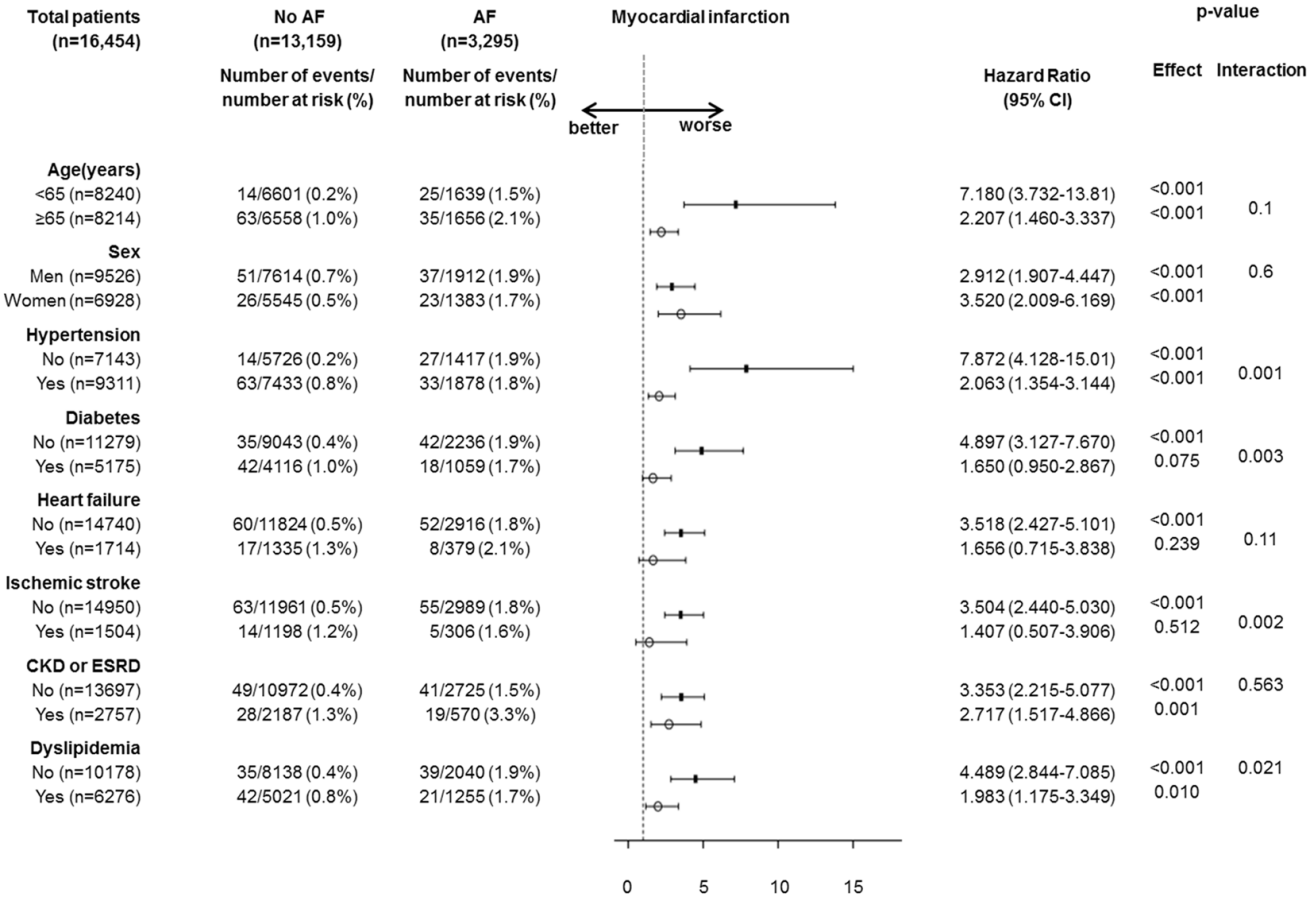
Effects of atrial fibrillation on the risk of myocardial infarction in different groups of patients. CI, confidence interval. Hazard ratios were calculated based on Cox regression after propensity matching. The P for interaction was calculated using the interaction term for AF and each subgroup based on Cox regression.

The associations of AF with MI among various subgroups of patients are displayed in Fig. 4. The risk of hospitalization or death due to MI was significantly higher in the AF group than in the no-AF group, regardless of age (p for interaction = 0.1), sex (p for interaction = 0.6), comorbidities with HF (p for interaction = 0.11), and CKD or ESRD (p for interaction = 0.56) at baseline. On the other hand, we found that the risk of AF associated incident MI was higher in patients free of hypertension [HR, 7.87 (95% CI, 4.13–15.0) vs. 2.06 (95% CI, 1.35–3.14), p for interaction = 0.001], diabetes [HR, 4.89 (95% CI, 3.13–7.67) vs. 1.66 (95% CI, 0.72–3.84), p for interaction = 0.003], ischemic stroke [HR, 3.50 (95% CI, 2.44–5.03) vs. 1.407 (95% CI, 0.51–3.91), p for interaction = 0.002], and dyslipidemia [HR, 4.49 (95% CI, 2.84–7.09) vs. 1.98 (95% CI, 1.18–3.35), p for interaction = 0.021] at baseline.

### Risk of MI in association with medications in AF patients

Medications analyzed in this study were aspirin, anticoagulants, statin, digoxin, BB and CCB. Anticoagulants included in the analysis were warfarin, direct thrombin inhibitor and factor Xa inhibitors. Pharmacological treatment was ascertained when there were at least 90 days or more prescriptions were prescribed after the incident diagnosis of AF.

**Figure 5:**
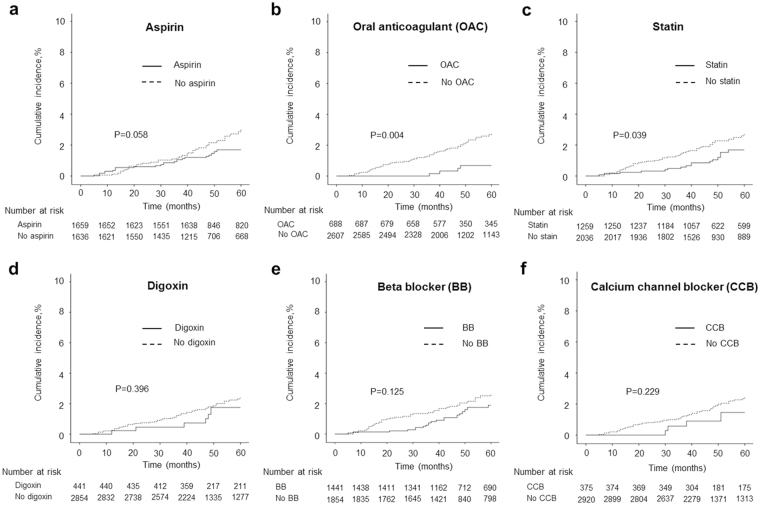
Risk of incident myocardial infarction associated with medication in patients with atrial fibrillation. The cumulative incidences of myocardial infarction were calculated using Kaplan-Meier estimates and compared using the log-rank test.

Figure 5 shows the effect of each medication on the occurrence of MI in AF patients. Anticoagulants were prescribed in 688 of the 3,295 AF patients without gender difference in prescription rate (men, 21.1% vs. women, 20.6%, p = 0.76). AF associated incident MI occurred less in patients on anticoagulant (0.6% vs. 2.1%, p = 0.004). Statins were prescribed in 1,259 of the 3,295 AF patients and prescription rate was higher in women than in men (men, 34.1% vs. women, 43.9%, p < 0.001). AF associated incident MI was less in patients on statin therapy (1.3% vs. 2.2%, p = 0.05). Cumulative incidence of MI was lower in patients who were taking an anticoagulant (Fig. 5b, p = 0.004) and statin (Fig. 5c, p = 0.039). Aspirin users showed a similar trend, which did not reach statistical significance (Fig. 5a, p = 0.058). The most commonly prescribed rate control agent was BB, followed by digoxin and CCB. AF-associated MI events did not differ between patients who were taking digoxin (Fig. 5d), BB (Fig. 5e), and CCB (Fig. 5f) and those who were not taking these medications. Overall death, regardless of the mode of death, occurred more often in patients with MI than in those without MI during the follow up period (35.8% vs 4.6%, p < 0.001).

## Discussion

In this study from NHIS, our principal findings are as follows: (1) AF was significantly associated with increased risk of incident MI. (2) Association between AF and the risk of MI did not differ by sex. (3) The risk of AF associated incident MI was higher in patients free of hypertension, diabetes, ischemic stroke, and dyslipidemia. (4) The incidence of AF associated incident MI was lower in patients on anticoagulant and statin therapies.

In this study, AF was associated with 3-fold increased risk of MI, even after the risk factors and potential confounders for MI were included in the matching. These findings were in accordance with the recently reported several studies which showed AF as an independent risk factor for MI^6,7,14,15^.

Contrary to recent studies which showed gender difference in the association between AF and MI^6,7,15^, it were not observed in this study. Previously, it was reported that women were less likely to receive warfarin than men^16,17^ and this may be the possible explanation for gender difference of MI^14^. However, in our study, use of anticoagulant did not differ between sex and this, in part, may contribute to the similar risk of MI between men and women.

Notably, we confirmed that the risk of MI was significantly higher in patients with AF free of overt cardiovascular disease than in subjects without AF. Previous study from Olmsted County revealed that lone AF had a comparable risk of embolic events and mortality to the general population^18,19^. In contrast, recent studies showed that patients with AF without cardiovascular co-morbidities had an increased risk of cardiovascular events and mortality^15,20^. These interactions suggest that shared underlying risk factors are not the only mechanisms explaining the association between AF and MI. There are plausible explanations for why AF patients with less co-morbidity were predisposed to MI. In patients with established risk factors for MI, AF may contribute less to the occurrence to MI, compared with those who were previously healthy. In our study, anticoagulants showed beneficial effect against AF associated incident MI. Accordingly, embolic events to coronary arteries are another possible explanation. Statin use, and also, is higher in patients with cardiovascular diseases, which may decrease the incident MI. Further study is needed in this regard.

In this analysis stratified by medications after AF diagnosis, the risk of incident MI was less in anticoagulant users. Previously, it was reported that warfarin might have a protective effect against MI^21^. Prevention of thrombus formation with anticoagulation, in part, might be an explanation. In our study, the anticoagulants analyzed were direct thrombin inhibitor, factor Xa inhibitors, as well as warfarin, and whether the new oral anticoagulants could have a role in preventing MI in the setting of AF needs to be further investigated.

Clinical trials have already demonstrated a clear beneficial effect of statin for prevention of ischemic heart disease^22,23^. In accordance with these studies, this study revealed that the risk of AF associated incident MI was less in statin users than in non-users. In a study, association between AF and inflammation has been reported^24^. The role of inflammation in MI has already been established^25^. Anti-inflammatory and anti-thrombotic activities of statin may reduce the risk of AF associated incident MI.

Type 2 MI can occur due to poorly controlled ventricular response in AF patients^26^. The rate control strategy in AF patients is expected to reduce the occurrence of type 2 MI, by reducing the oxygen demand and by increasing the diastolic filling time of coronary arteries. However, the result of this study was not in accordance with this common belief, suggesting that type 2 MI is less likely to be the main mechanism by which AF leads to MI.

Considering MI as a known risk factor for AF^27^, this result suggests a bidirectional relationship between AF and MI. However, the pathophysiology of AF associated incident MI is complex and incompletely understood. Further research to determine the underlying mechanism with the association of AF and MI is warranted. The prevalence of AF doubles with each additional decade of life^28^. With an increasing older population, the occurrence of AF itself and AF-associated morbidity and mortality is expected to increase as well. From a preventive perspective, the notion should suggest that AF potentiates the risk of MI.

## Limitations

There are several limitations to this study. Although we ascertained AF cases with ECGs as well as ICD codes, it is possible that paroxysmal or asymptomatic AF cases were not detected. Also, we could not analyze paroxysmal, persistent and permanent AF separately. Second, because MI cases were identified by ICD-10 code, data for cardiac markers, ECGs, and coronary angiography were not available, which could have provided further information about the type of MI^26^. We could not analyze the association of AF with different types of MI. Third, even though the NHIS recommends biennial health check-up to all health insurance subscribers, it is not compulsory and there is a possibility that healthier and more educated subjects could have participated in this study. Fourth, potentially uncontrolled covariates may exist because comorbidities included in propensity matching are the data from baseline examination. They may be absent at baseline, but may have developed during the follow-up. Fifth, similar to other studies, considerable confounders may remain although propensity-score matching was well balanced. Despite the limitations, this study is the nation-wide population based observational studies providing the further evidence for a link between AF and MI, and the effects of medications on the occurrence of AF related incident MI.

## Conclusions

AF is associated with an increased risk of MI in the nationwide cohort study of Korean population. The association between AF and MI did not differ by sex and higher in patients free of cardiovascular diseases at baseline. Anticoagulants and statin showed beneficial effects against MI in AF patients. Our finding suggests that AF complications beyond stroke should extend to total mortality to include MI.

## Electronic supplementary material

Supplementary information
