# Supplementary material for: Atrial fibrillation and the risk of myocardial infarction: a nation-wide propensity-matched study
Source: Sci Rep. 2017 Oct 5;7:12716. doi: 10.1038/s41598-017-13061-4 (PMC5629219; doi:10.1038/s41598-017-13061-4)
Supplement: Supplementary file 1 — Supplementary information [file 41598_2017_13061_MOESM1_ESM.pdf]

Supplementary information

Atrial fibrillation and the risk of myocardial infarction: a nation-wide propensity-matched study

Hye Young Lee,[1 2] Pil-Sung Yang,[1] Tae-Hoon Kim,[1] Jae-Sun Uhm,[1] Hui-Nam Pak,[1] Moon-Hyoung Lee,[1] Boyoung Joung[1]

[1]Division of Cardiology, Yonsei University College of Medicine, Seoul, Korea, [2]Division of Cardiology, Sanggye Paik Hospital, Inje University College of Medicine, Seoul, Korea

Table S1. International Classification of Disease 10th codes for comorbidities

**Diagnosis of Comorbidities**		
Atrial fibrillation	Defined from diagnosis*	ICD10: I48
Ischemic stroke	Defined from diagnosis*	ICD10: I63, I64
Heart failure	Defined from diagnosis*	ICD10: I11.0, I50, I97.1
Diabetes mellitus	Defined from diagnosis*	ICD10: E10, E11, E12, E13, E14
Hypertension	Defined from diagnosis*	ICD10: I10, I11, I12, I13, I15
Myocardial infarction	Defined from diagnosis*	I21, I22, I25.2
Peripheral arterial obstructive disease	Defined from diagnosis*	ICD10: I70.0, I70.1, I70.2, I70.8, I70.9, I73.9, I79.2
Dyslipidemia	Defined from diagnosis*	ICD10: E78
Chronic obstructive lung disease	Defined from diagnosis*	J42, J43(except J43.0), J44
Chronic renal failure	Defined from eGFR	eGFR <60 mL/min per 1.73 m^2
End state renal disease	Defined from national registry for severe illness.	Patients with ESRD undergoing ch ronic dialysis or received a kidney transplant.
Malignancy	Defined from diagnoses of cancer (non-benign)	ICD10: C00-C97
Potential absence of non-valvular atrial fibrillation	Defined from any diagnoses or operation of mitral stenosis	ICD10: I05.0, I05.2, I34.2, Z95. 2-4
Chronic Liver disease	Defined from diagnosis of chronic liver disease, cirrhosis, and hepatitis	ICD10: B18, K70, K71, K72, K73, K74, K76.1

*To ensure accuracy, comorbidities were established based on one inpatient or two outpatient records of ICD-10 codes in the database.

Figure S1. Love plots for absolute standardized difference for baseline covariate between patients with and without atrial fibrillation, before and after propensity score matching

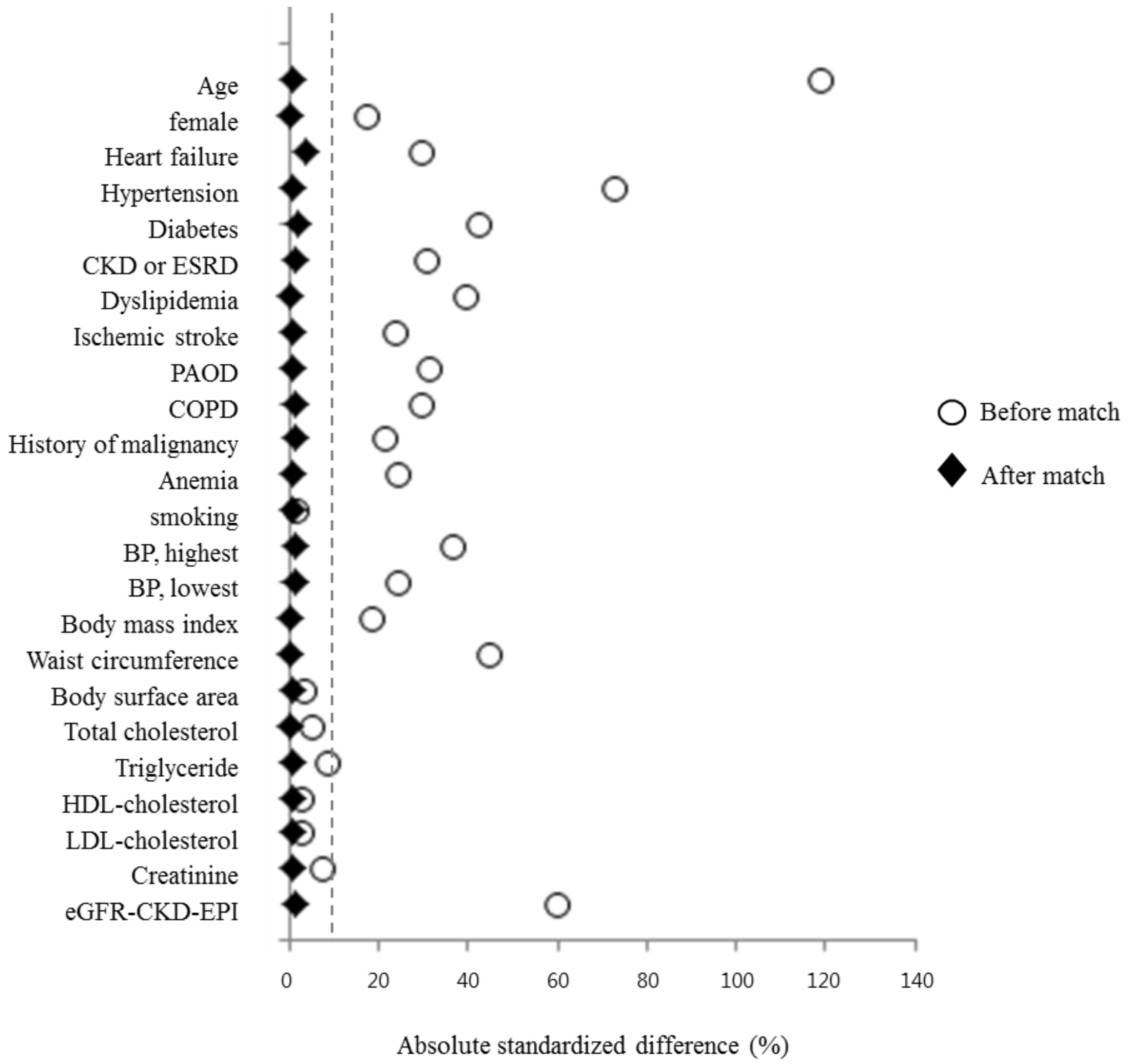

CKD, chronic kidney disease; ESRD, end–stage renal disease; PAOD, peripheral artery obstructive disease; COPD, chronic obstructive pulmonary disease; BP, blood pressure; HDL, high-density lipoprotein; LDL, low-density lipoprotein; eGFR-CKD-EPI, estimated glomerular filtration rate-chronic kidney disease-epidemiology collaboration
